# The Loss of Metabolic Control on Alcohol Drinking in Heavy Drinking Alcohol-Dependent Subjects

**DOI:** 10.1371/journal.pone.0038682

**Published:** 2012-07-09

**Authors:** Philippe de Timary, Patrice D. Cani, Julie Duchemin, Audrey M. Neyrinck, Dominique Gihousse, Pierre-François Laterre, Abdenor Badaoui, Sophie Leclercq, Nathalie M. Delzenne, Peter Stärkel

**Affiliations:** 1 Department of Adult Psychiatry, Saint-Luc Academic Hospital and Psychological Development (CSDP) Research Unit, Institute of Neuroscience and Institute of Psychology, Catholic University of Louvain, Brussels, Belgium; 2 Metabolism and Nutrition Research Group, Louvain Drug Research Institute, Catholic University of Louvain, Brussels, Belgium; 3 Department of Dietetics, Saint-Luc Academic Hospital, Catholic University of Louvain, Brussels, Belgium; 4 Department of Anestesiology, Saint-Luc Academic Hospital and Institute of Clinical Research, Catholic University of Louvain, Brussels, Belgium; 5 Department of Gastroenterology, Saint-Luc Academic Hospital and Institute of Clinical Research, Catholic University of Louvain, Brussels, Belgium; University of Cordoba, Spain

## Abstract

**Background:**

Most physiological studies interested in alcohol-dependence examined ethanol as a pharmacological agent rather than a nutrient. We conducted two studies, which assessed the metabolic and endocrine factors involved in the regulation of alcohol and nutrient intake in alcohol-dependent (AD) subjects. We also examined the potential role of a disruption in energy balance in alcohol-dependence.

**Methods and Results:**

In Study-1, quantitative dietetic interviews of eating and drinking habits were conducted with 97 AD subjects. The population was split around a median alcohol intake value of 12.5 kcal/kg/day. The results showed that the “low alcohol” drinking AD subjects had high Body Mass Index (BMI) and Fat Mass (FM) and alcohol intake was compensated for by a decrease in non-alcoholic intakes. “High alcohol” drinking AD subjects, on the other hand, had low BMI and FM and the total caloric intakes were largely above norms. In Study-2, 24 AD inpatients were submitted to dietetic interviews, calorimetry and blood samplings for the measurement of biomarkers of the regulation of metabolism and satiety, on day 2, 5 and 16 of abstinence. These patients were compared with 20 controls matched for age and gender. We observed in AD patients an increase in cortisol, leptin and PYY plasma levels and a decrease in ghrelin, which might explain the observed decrease in non-alcoholic intakes. However, alcoholic and non-alcoholic intakes correlated positively with basal metabolism and negatively with leptin and leptin/BMI.

**Conclusion:**

For individuals consuming below12.5 kcal/kg/day of alcohol, alcohol intake is compensated for by a decrease in non-alcoholic nutrient intakes, probably due to changes in metabolic and satiety factors. For individuals consuming above 12.5 kcal/kg/day of alcohol, alcohol accelerates metabolism and decreases fat mass and leptin levels, and the total caloric intake largely exceeds norms. A dual model for regulation of energy intake in AD subjects is proposed.

## Introduction

“Where the brewer passes, the baker does not.” This popular Belgian quotation alludes to the fact that alcohol-dependent (AD) subjects, or at least those who drink beer, often replace food consumption with substantial alcohol consumption. Previous epidemiological studies have indeed shown that alcohol may account for 10% of total energy consumption in alcohol consumers and for more than 50% of dietary intake in AD subjects [1]–[5]. However, alcohol-dependence, which is present in 5 to 7% of the population in developed countries, is associated with weight gain but it is also considered as a leading cause of malnutrition, suggesting a complex relation between eating and drinking habits in these subjects. Indeed, several studies have reported a positive relation between alcohol consumption and body weight [6]–[8], whereas others have reported a negative one [9]–[12] or no relation at all [13]–[16]. One of possible explanations for these discrepant results is that some studies reported the relation in normal populations, whereas others described AD subjects presenting, or not, additional illnesses. The relation between alcohol and food consumption, and how both are related to body-weight, fat mass or metabolism in AD subjects has only been studied quantitatively in a few prior studies. Furthermore, these studies have only selected patients with very high alcohol intakes [17] or individuals who were hospitalized for additional illnesses [18] which may have also influenced body mass and appetite.

Recently, the peptides that influence body and fat mass and present anorexigenic or orexigenic properties have been studied in relation to alcohol consumption. With regard to leptin, Mantzoros et al. [19] showed that obesity and alcohol intake were both positively associated with circulating leptin in young healthy men, whereas other studies failed to observe a relation between leptin and alcohol intake [20], [21]. For example, Santolaria et al. [18] found that AD subjects who drink more than 80 g ethanol/day exhibit decreased levels of fat mass and leptin.

Finally, several researchers have observed a positive correlation between alcohol craving and plasma concentrations of the anorexigenic peptides leptin [22]–[24], insulin [25], and orexigenic peptide ghrelin [26], [27]. These observations suggest that a relation exists between the regulation of food and alcohol intake. However, the role that (an)orexigenic peptides play in the modulation of metabolic and/or nutritional control of alcohol consumption remains unexplored.

Thus, the aim of the present work was to analyse, quantitatively, energy metabolism in AD subjects who vary in levels of alcohol consumption. Indeed, unlike prior research which only selected AD subjects who consumed very high quantities of ethanol, we decided to select all populations of AD subjects. We also excluded all subjects that presented additional illnesses, which may also affect appetite and energy regulation. For this project, we conducted two studies.

The first consisted of careful diet recall interviews with 97 AD inpatients to assess: 1- the importance of alcohol-related or unrelated energy supplies and how both are interrelated; 2- the relation between alcohol and non-alcoholic nutrients intake and Body Mass Index (BMI) and Fat Mass (FM).

A second metabolic study was performed on 24 AD subjects matched for age and gender with 20 control subjects, in order to examine the regulatory mechanisms of energy balance during alcohol-withdrawal. Therefore, at the onset, during, and at the end of the withdrawal, we measured alcoholic and non-alcoholic intake, basal metabolism and morphometric parameters including BMI and FM. We also assessed various anorexigenic and orexigenic peptides, which are potent regulators of the energy balance (leptin, insulin, cortisol) and of the meal duration (ghrelin, peptide YY (PYY), glucagon-like peptide 1 (GLP-1)) [28].

Finally, we designed a new comprehensive model to explain the regulation of food and alcohol intakes in AD subjects.

## Methods

### Ethics Statement

The study protocol was approved by the ethical committee of the hospital (Commission d’éthique biomédicale hospitalo-facultaire, UCL) and all subjects signed an informed consent form prior to the investigation.

### Subjects and Procedures

A total of 97 inpatients admitted for alcohol withdrawal and rehabilitation, were enrolled in **study-1** during a 12-month period. All patients fulfilled the DSM - IV criteria of alcohol-dependence [29]. The exclusion criteria were as follows: psychiatric diseases other than alcohol-dependence, clinical or laboratory evidence of liver cirrhosis, diabetes mellitus, glucose intolerance, pancreatic disease and other diseases which may affect appetite and energy regulation. Among this group of 97 inpatients of study-1, 33 subjects who had been drinking until day of admission were included in **study-2** and tested for metabolic, endocrine, and psychological parameters between their admission and day 16. Of these, 9 were excluded for having left the rehabilitation programme. The remaining 24 subjects were compared to 20 control social drinkers matched for age and gender.

#### Procedure for study-1

Patients were interviewed by expert dieticians concerning periods of active alcohol drinking during the 7 days that preceded hospital admission. This dietary recall interview [30], was collected by two expert dieticians (DG and JD), using the hospital’s standardized semi-structured paper and pencil retrospective interview form, adapted to alcohol-dependent populations according to the principles of the time-line followback approach [31], [32] (see supplemental material and methods section Text S1).

#### Procedure for study-2

Between 8 and 9 AM on days 2 (T1), 5 (T2) and 16 (T3) of withdrawal, patients underwent fasting blood samples to determine their plasma glucose, insulin, cortisol, leptin, ghrelin, Peptide YY (PYY) and Glucagon Like Peptide-1 (GLP-1) levels. Aspartate aminotranferase (AST), Alanine aminotransferase (ALT), γ-Glutamyl transpeptidase (γ-GT), total bilirubin, total blood protein (TBP), albumin and mean corpuscular volume (MCV) were measured on day 2. On days 2 and 16, before blood sampling, the patients were also subjected to indirect calorimetry, a thorough food interview regarding the previous 7 days, and a bioelectric impedance measurement of fat mass (FM). The methodology is detailed in Text S1.

### Statistical Analysis

#### Study 1

Due to the large sample size (n = 97), we assumed statistical normality for each variable. Independent student’s t tests were used to compare female AD vs. male AD, “low alcohol” drinking AD vs. “high alcohol” drinking AD, and AD patients vs. controls.

#### Study 2

The comparison of means at T1, T2 and T3 was tested with one-way repeated-measures ANOVA. Data that exhibited a non-normal distribution were log-transformed to obtain normality. The assumption of sphericity, assessed with the Mauchly’s test, was met for each variable (*p*>0.05). When the results of ANOVA were significant (p<0.05), *post hoc* tests with Bonferroni adjustment for multiple comparisons were used for pairwise comparisons. Finally, we conducted independent Student’s *t-tests* to compare AD patients with the control group. Correlations between scores of variables were calculated using Pearson moment coefficients for linear relations and Spearman coefficients for non-linear ones.

## Results

### Study 1. Interplay between Alcoholic and Non-alcoholic Intakes and their Relation to BMI and Fat Mass in AD Subjects

The characteristics of the population of study 1 are reported in Table 1. Male and female populations evidenced similar BMI, but the FM was significantly higher for females. The mean alcohol intake was 159.4±108.7 g per day (range 32–587).

**Table 1:** Characteristics of AD subjects in study 1.

	Males (n = 63)	Females (n = 34)	Males + Females (n = 97)
**Anthropomorphic parameters**			
Age (y)	49±12	49±10	49±11
Weight (kg)	77.7±15.1	66.4±13.8***	73.7±15.6
BMI (kg/m^2^)	24.6±4.3	24.6±5.0	24.7±4.5
FM (%) (M; n = 31/F; n = 14)	25.3±8.4	33.9±6.9**	28.0±8.9
BM theoretical (kcal)	1743.9±193.3	1402.4±162.0***	1624.2±244.9
**Nutrient intakes (kcal/kg/day)**			
Total	42.7±21.0	32.2±12.7**	39.0±19.1
Proteins	3.6±1.7	3.5±1.4	3.5±1.5
Lipids	7.6±4.3	6.7±3.6	7.3±4.1
Carbohydrates	14.1±9.6	10.6±5.5*	12.9±8.5
Non-alcohol	25.3±14.8	20.2±8.7	23.5±16.0
Alcohol	17.4±11.5	12.0±8.1**	15.5±10.7
**Nutrient intakes (%)**			
Proteins	9.4±3.4	10.7±4.0*	9.8±3.7
Lipids	19.0±8.1	20.3±8.8	19.5±8.3
Carbohydrates	32.0±9.7	30.7±9.8	31.6±9.7
Non-Alcohol	60.1±13.8	64.7±12.8	61.7±13.6
Alcohol	39.2±14.1	38.3±16.6	38.9±14.9

Values are means ± SD.

* p<0.05,

** p<0.01,

*** p<0.001 (Males vs Females).

BMI: body mass index; FM: fat mass; BM: basal metabolism.

#### Importance and interrelation of alcoholic and non-alcoholic energy supplies

From the dietary interviews, we calculated the number of meals taken per 7 days for each participant. It was significantly lower for breakfast (2.72±3.06) than for lunch (4.31±2.79) or for dinner (6.09±2.02) and these proportions were not significantly different across males and females. The nutrient and alcohol (ethanol) intakes are presented in Table 1. Ethanol consumption accounted for 38.9±14.9% of total energy supplies (range = 12–68%) in AD subjects. The total caloric intake, carbohydrates and alcohol intakes (expressed as kcal/kg/day) were lower in females than in males. The proportion of the different macronutrients remained similar in males and females except for the percentage of protein intake that was higher in females. When we compared AD subjects according to the alcohol type (Beer – Wine – Spirit), we found that alcohol intake but also non-alcohol and total caloric intakes were significantly higher for beer drinking AD subjects. This was due to an increase in starch intake in beer drinking subjects, whereas protein and lipid intakes remained similar between groups (see Table S1). We also observed a lower ethanol intake in wine drinking subjects, compared to the two other groups.

**Figure 1:**
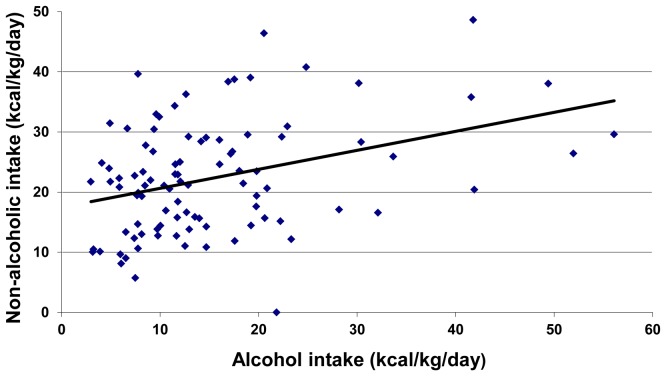
Relation between alcoholic and non-alcoholic intakes in AD subjects.

We then split our 97 subjects population into two (“low alcohol” vs. “high alcohol” drinking AD subjects) groups based on the median value of alcohol intake (12.5 kcal/kg/day). The “low alcohol” group evidenced a higher body weight, a higher BMI and a higher proportion of FM than the “high alcohol” group (Table 2). However, the proportion of females was higher in the “low alcohol” group. This could explain the increase in FM observed in the “low alcohol” group, fat being higher in women than in men. We therefore split our population according to gender (male vs. female) in both groups (low vs. high alcohol). We observed that men exhibited higher weight, BMI and FM in the “low alcohol” group compared with the “high alcohol” group. In women, weight was also significantly higher in the “low alcohol” group, but the observed differences in BMI and FM were not significant between groups. However, this may be due to the low number of women in the “high alcohol” group, thereby preventing us from making definitive interpretations. Figure 1 shows a positive relation between alcoholic and non-alcoholic supplies (Pearson *r* = 0.36, *p*<0.001) and huge differences in energy intake within the population of AD subjects.

**Table 2:** Characteristics and intakes for AD subjects drinking “low” or “high” quantities of alcohol.

	“Low alcohol” (n = 48)	“High alcohol” (n = 49)
**Anthropomorphic parameters**		
Age (y)	51±10	47±12
Gender (male/female)	24/24	39/10
Weight (kg)	76.6±15.7	71.0±14.9
*- Male (low; n = 24/high; n = 39)*	*83.2±16.0*	*74.3±13.6* *
*- Female (low; n = 24/high; n = 10)*	*70.0±12.9*	*57.8±12.7* *
BMI (kg/m^2^)	26.1±4.6	23.2±4.0**
*- Male (low; n = 24/high; n = 39)*	*26.8±4.3*	*23.4±3.9* **
*- Female (low; n = 24/high; n = 10)*	*25.7±4.9*	*22.3±4.5*
FM (%) (low; n = 21/high; n = 24)	32.5±7.8	24.0±7.9***
*- Male (low; n = 10/high; n = 21)*	*30.5±7.9*	*22.9±7.6* *
*- Female (low; n = 11/high; n = 3)*	*34.4±7.6*	*32±4.1*
**Nutrient intakes (kcal/kg/day)**		
Total	28.1±9.0	47.5±17.3***
Proteins	3.2±1.3	3.5±1.4
Lipids	7.0±3.3	6.7±3.4
Carbohydrates	9.6±4.6	14.5±8.0***
Non-alcohol	19.8±7.8	24.7±10.4**
Alcohol	8.3±2.7	22.8±10.8***

Values are means ± SD.

* p<0.05,

** p<0.01,

*** p<0.001 (“low alcohol” vs “high alcohol”).

The total population of alcoholics was split into two subpopulations depending on whether their consumption was lower or higher than the median value of 12.5 kcal/kg/day of alcohol and described as “low alcohol” or “high alcohol” drinking alcoholics. BMI: body mass index; FM: fat mass.

#### The relation between energy intakes, BMI and FM

**Figure 2:**
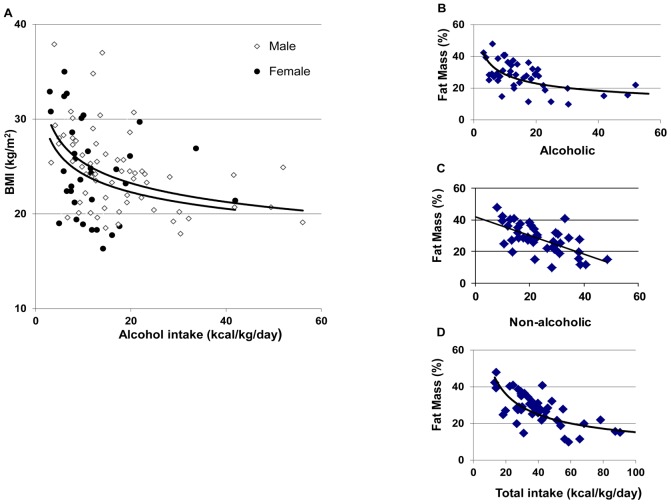
Relation between alcohol intake and BMI (A) and between intakes and fat mass (B,C,D).

When examining the relation between BMI and alcohol intake we, surprisingly, observed a negative correlation for both male and female subjects (Fig 2A). The best fits were obtained for curvilinear power relations. The Spearman correlation coefficients (*r*) were -0.38 (*p*<0.0001), -0.48 (*p*<0.0001) and -0.36 (*p*<0.05) for the total, the male, and the female populations, respectively. We also observed a negative correlation between BMI and non-alcoholic intake with Spearman *r’s* of -0.37 (*p*<0.001), -0.38 (*p*<0.01) and -0.38 (*p*<0.05) for the total, the male, and the female populations, respectively. A similar curvilinear, negative relation was also found between FM and alcohol intake (Fig 2B) and total intake (Fig 2D), the Spearman *r’s* were -0.51 and -0.61, *p*<0.001, respectively. We also observed a negative correlation between FM and non-alcoholic nutrient intake but the best fit was a linear relation, Pearson *r* = −0.63, *p*<0.001 (Fig 2C).

### Study 2. Evolution of Alcoholic and Non-alcoholic Intakes, Metabolism and Orexigenic and Anorexigenic Peptides during Alcohol Withdrawal

A group of 24 AD subjects who had drank alcohol until the day of admission was compared to a group of 20 controls matched for age and gender**.** The control group was tested once, and the AD subjects were tested on days 2 (T1), 5 (T2) and 16 (T3) of alcohol withdrawal.

Table 3 shows the general and metabolic characteristics of both groups. The indexes of liver and synthetic function of this population are given as supplemental material (Table S2).

**Table 3:** Anthropomorphic, calorimetric and meal characteristics of AD and control subjects in Study 2.

	AD-T1 (n = 24)	AD-T3 (n = 24)	Controls (n = 20)
**Anthropomorphic parameters**			
Age	47±10	47±10	44±9
Gender (Male/Female)	15/9	15/9	13/7
Weight (kg)	72.8±12.8	73.2±13.2	75.2±14.1
BMI (kg/m^2^)	24.6±3.9	24.7±4.0	24.2±3.0
FM (%)	27.6±9.1*	25.9±9.4##	25.3±7.5
Waist size (cm)	93.0±11.4	92.0±11.1	91.0±11.7
BM (theoretical) (kcal)	1617.0±224.3	1621.7±230.7	1667.8±267.6
BM (calorimetry) (kcal)	1699.1±285.5	1549.1±274.4###	1674.3±299.0
Respiratory Quotient	0.791±0.057**	0.843±0.051##	0.838±0.054
**Meal frequency (/7 days)**			
Breakfast	3.0±3.3***	4.9±3.2#*	7.0±0.0
Lunch	5.1±2.4*	6.3±1.4#	6.5±1.0
Dinner	6.8±0.7	6.8±0.7	7.0±0.0

Values are means ± SD.

* p<0.05,

** p<0.01,

*** p<0.001 (AD vs Controls).

# p<0.05,

## p<0.01,

### p<0.001 (AD-T1 vs AD-T3).

Theoretical basal metabolism was calculated according to the Schofield equations. Respiratory Quotient was calculated as CO_2_ consumed/O_2_ output. BMI: body mass index; FM: fat mass; BM: basal metabolism.

At the onset of withdrawal, the AD subjects evidenced a similar weight, BMI and waist size and theoretical basal metabolism compared to controls. The FM calculated by impedancemetry was higher in AD subjects than in controls and decreased significantly during withdrawal to values similar to the control values. Basal metabolism, calculated by calorimetry, was similar to that of controls at onset of withdrawal in AD subjects, but it decreased significantly during withdrawal. In AD subjects, at T1, the respiratory quotient (RQ) was significantly lower than that of controls and it increased significantly during withdrawal (Table 3).

#### The influence of alcohol intake and withdrawal on nutrient intake

We observed a large decrease in meal frequency at breakfast and lunch in AD subjects at T1 compared with the controls (Table 3). However, at T3, breakfast and lunch frequency had partially or totally recovered, respectively. From a quantitative standpoint, alcohol accounted for 36% of total energy intake of AD subjects (Table 4). Due to these consequential alcoholic supplies, the total caloric intakes were higher than that of controls whereas the non-alcoholic intakes were significantly lower. At the end of the withdrawal, we observed a significant increase in non-alcoholic nutrient intake (in terms of kcal/kg/day) but this intake remained largely lower than that of controls. In AD subjects, the protein and carbohydrate intakes remained unchanged but the lipid intake increased significantly from T1 to T3. After withdrawal, the proportions of energy supplied by each macronutrient were similar to those of controls (Table 4).

**Table 4:** Nutrients intakes in controls and AD subjects at the onset (T1) and end (T3) of withdrawal.

	AD-T1 (n = 24)	AD-T3 (n = 24)	Controls (n = 20)
**Nutrient intakes (kcal/day)**			
Total	2859.0±1118.3	1913.0±456.2***	2404.4±761.4¶
Proteins	282.2±63.4	282.8±63.8	367.9±88.5** ¶¶
Lipids	599.0±196.9	707.3±210.2*	849.7±313.5**
Carbohydrates	893.7±434.1	906.0±302.6	1084.9±362.3
Non-alcohol	1773.5±586.7	1891.5±462.2	2304.6±716.1* ¶
Alcohol	1085.5±704.1	21.5±51.0***	103.7±96.8*** ¶¶
**Nutrient intakes (kcal/kg/day)**			
Total	39.9±15.9	27.1±9.3***	31.8±7.2*
Proteins	4.0±1.1	4.0±1.2	4.9±0.8* ¶
Lipids	8.5±3.2	10.1±4.0*	11.1±2.7*
Carbohydrates	12.3±6.1	12.7±5.4	14.5±4.3
Non-alcohol	24.9±8.6	26.8±9.4*	30.5±7.0* ¶
Alcohol	15.0±10.0	0.3±0.6***	1.4±1.2*** ¶¶
**Nutrient intakes (%)**			
Proteins	10.7±3.1	15.0±2.8***	15.6±2.3***
Lipids	22.5±8.4	37.2±8.4***	34.9±4.1***
Carbohydrates	30.6±8.5	46.7±10.2***	45.2±4.4***
Alcohol	36.2±11.5	1.2±2.8***	4.3±3.4*** ¶¶
**Protein intakes (g/kg/day)**	0.99±0.27	1.00±0.31	1.22±0.20** ¶

Values are means ± SD.

* p<0.05.

** p<0.01.

*** p<0.001 (vs AD-T1).

¶ p<0.05.

¶¶ p<0.01 (vs AD-T3).

#### The relation between nutrient intakes, basal metabolism and orexigenic and anorexigenic peptides

**Figure 3:**
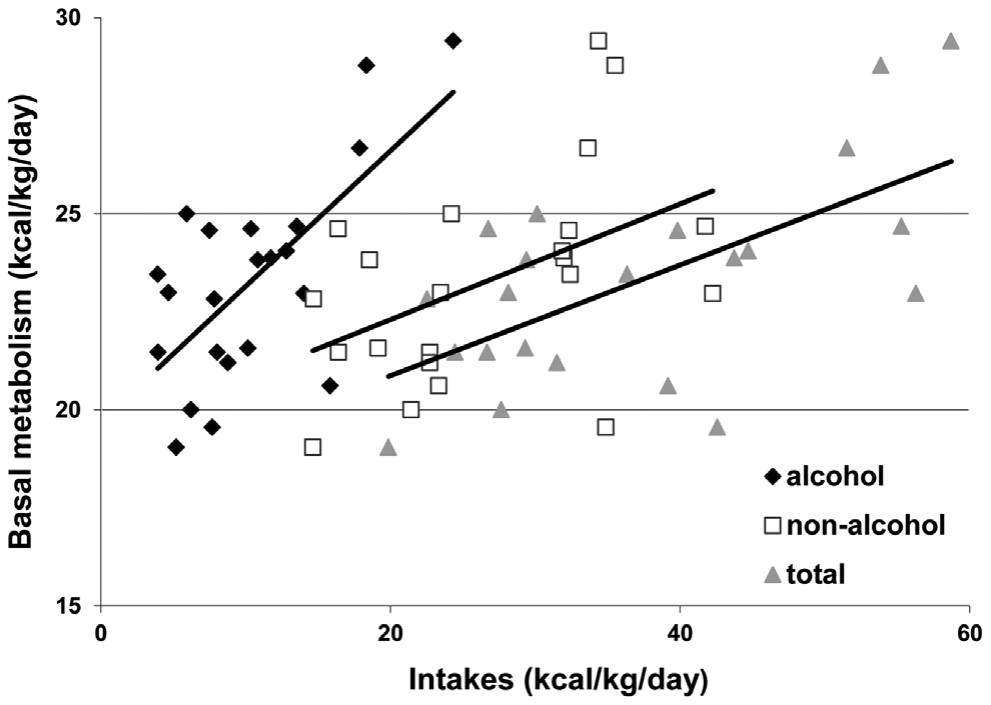
Relation between basal metabolism and intakes at the onset of withdrawal.

Figure 3 shows, at onset of withdrawal, a strong positive correlation between basal metabolism measured by calorimetry and alcoholic (*r* = 0.69, *p*<0.001), non-alcoholic (*r* = 0.47, p<0.05) or total energy intakes (r = 0.63, p<0.005). We next evaluated the evolution of several orexigenic and anorexigenic peptides during withdrawal and compared them to the values obtained in controls (Table 5). The leptin and leptin/BMI were significantly higher compared to controls at T2 and marginally higher at T1. Conversely, the plasma ghrelin levels at T1 and T2 were significantly lower and the PYY levels at T1, T2 and T3 were significantly higher compared to controls. The GLP-1 levels were significantly lower at T2 and T3 compared to controls. At T2, the insulin levels were significantly higher than controls. Insulin sensitivity calculated according to the HOMA model was decreased at T2 compared to T1. Insulin secretion calculated according to the HOMA model was increased at T2 and T3 compared to controls. Cortisolemia was higher at onset of withdrawal and decreased progressively but the recovery was only partial compared to controls.

**Table 5:** Plasma concentrations of (an)orexigenic peptides, glucose and cortisol, estimation of insulin sensitivity and secretion using the HOMA model in AD subjects during withdrawal and controls.

	AD-T1 (n = 24)	AD-T2 (n = 24)	AD-T3 (n = 24)	Controls (n = 20)
Leptin (µg/L)	7,7±5,3	8,6±6.0	7.0±5,5§§	5,1±3.7§
Leptin/BMI	0.30±0,17	0.33±0.20	0.27±0.18§§	0.21±0.15§
Ghrelin (pg/ml)	102.0±34.4	99.3±35.5	104.6±39.8	130.5±48.9#§
PYY (pg/ml)	110.9±37.3	110.1±31.5	113.8±38.7	91.5±21.4#§ ¶
GLP-1(pm/ml)	2.48±1.57	2.24±1.56	2.23±1.56	3.2±1.2§ ¶
Insulin (µU/ml)	3.8±1.6	5.2±2.8##	4.5±2.5	3.3±1.4§
Glycemia (mg/dl)	86.8±10.7	79.1±10.2#	83.5±13.2	84.6±8.1
HOMA %S	187.4±70.8	151.9±67.3#	178.4±82.0	200.7±83.9
HOMA %B	73.4±21.3	113.6±57.5#	85.6±25.3#§	70.9±15.6§ ¶
Cortisol (ng/ml)	447.5±154.4	419.7±131.4	350.2±139.4#§	234.3±57.6###§§§ ¶¶

Values are means ± SD.

# p<0.05,

## p<0.01, p<0.001 (compared to AD-T1).

§ p<0.05,

§§ p<0.01,

§§§ p<0.001 (compared to AD-T2).

¶ p<0.05,

¶¶ p<0.01 (compared to AD-T3).

**Figure 4:**
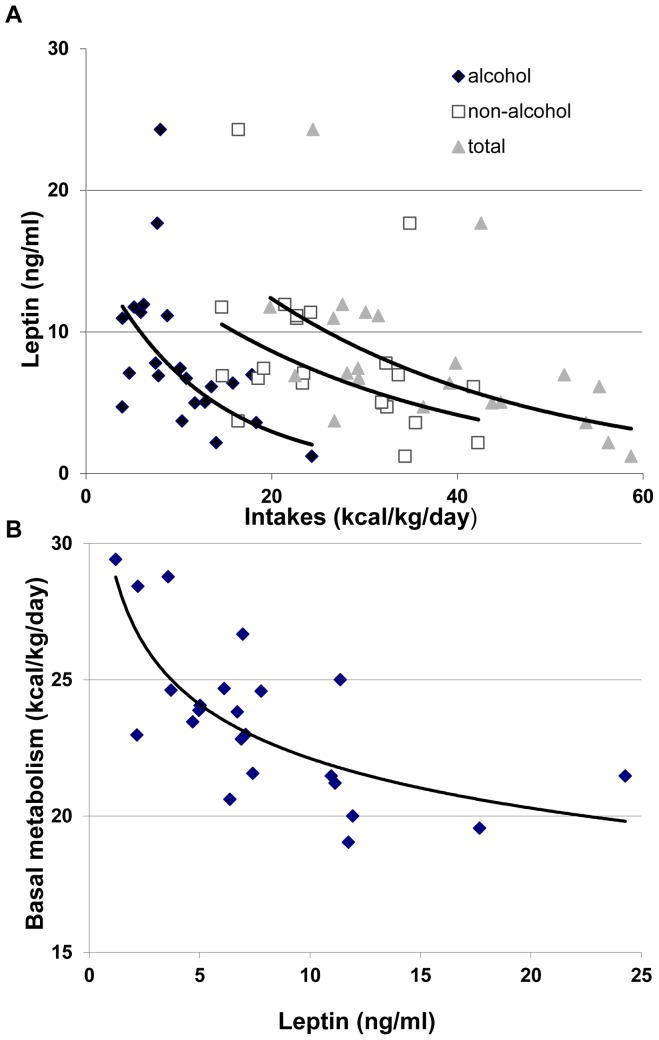
Relation between leptinemia and intakes (A) and between leptinemia and basal metabolism (B).

We next calculated correlations between nutrient intakes and the different orexigenic and anorexigenic factors. All the correlations were non-significant except for a negative correlation between alcoholic, non-alcoholic or total energy intakes and leptin, with Spearman r coefficients of -0.61 (p<0.005), -0.44 (p<0.05) and -0.59 (p<0.005), respectively (Fig 4A). The leptin/BMI were also negatively correlated to alcoholic, non-alcoholic or total energy intakes with Spearman r coefficients of -0.63 (p<0.005), -0.52 (p<0.01) and -0.66 (p<0.005) respectively. We also observed a negative correlation between leptin and basal metabolism calculated by calorimetry, with Spearman coefficient of -0.62 (p<0.0001) (Fig 4B).

**Figure 5:**
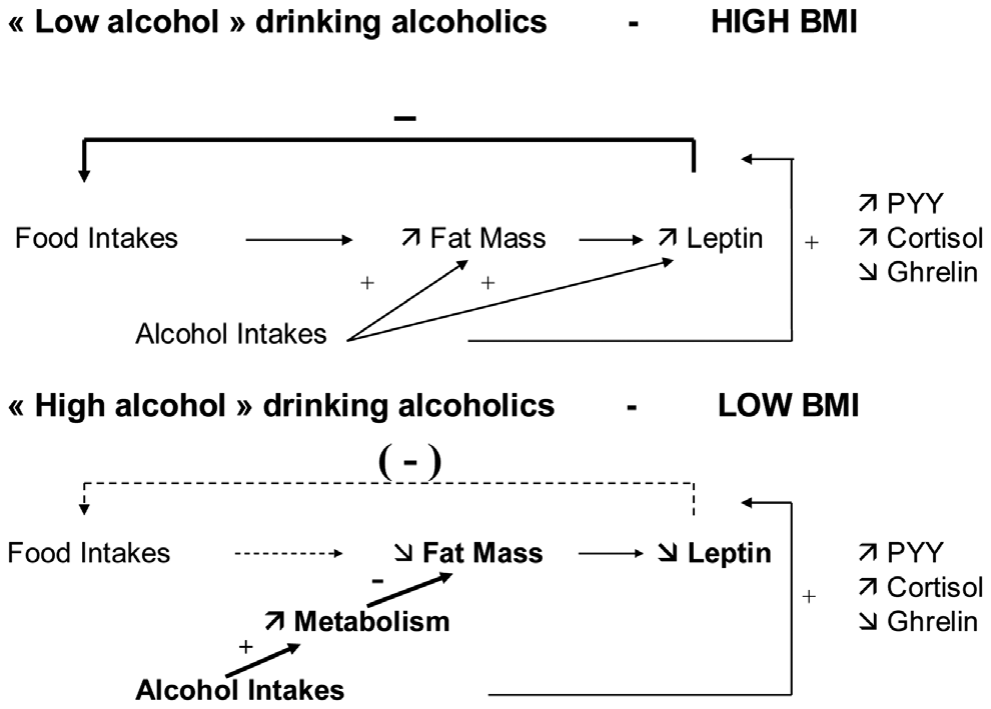
Model for disruption of energy balance in AD subjects.

## Discussion

This article focused on energy balance in alcoholic patients before and after alcohol withdrawal. All the patients diagnosed with alcohol-dependence according to the DSM-IV criteria were included in the study and the quantity of ethanol consumed daily did not serve as an exclusion criteria. This permitted the observation of subjects with large ranges of alcohol consumption in their daily diet.

### The Relation between Eating and Drinking Habits in AD Subjects

Our methodology was based on a dietary recall interview [30], adapted to alcohol-dependent populations according to the principles of the time-line followback approach [31], [32]. This methodology consists of a retrospective face-to-face interview on the 7 days that preceded admission to the hospital, by expert dieticians. Indeed, it was shown that the use of prospective self-reported dairies is often associated with a risk of under-eating in periods of recording [33], [34]. Also, self-reported questionnaires generally lead to under-reporting, especially in individuals with high BMI [35] and heavy alcohol consumption can lead to inappropriate answers to questionnaires. In our 7-day dietary recall interview, the questions are posed in a redundant manner in order to ensure the quality of our results.

From a phenomenological standpoint, we observed a specific modification of the eating habits in AD subjects, with a large decrease in frequency of having breakfast or lunch but a normal frequency of having dinner. Changes in eating habits of AD subjects have previously been correlated with disorganisation of living and social habits [36]. In the second week of abstinence, we observed a recovery of non-alcoholic intakes, which was partial when compared to controls. Quantitatively, the non-alcoholic energy supplies were lower for active drinking AD subjects compared to the norms, especially for lipids and carbohydrates. Indeed, according to the Belgian dietary guidelines, the intake of proteins, lipids and carbohydrates represent respectively 0.8 g/kg/day, a maximum of 30–35% of total energy intake, and at least 55% of total energy intake [37]. Alcoholic supplies were compensated for by a decrease in non-alcoholic supplies in females and only partially in males. However, surprisingly, AD subjects exhibited a positive relation between alcoholic and non-alcoholic energy supplies. In an attempt to resolve this paradox, we split our population around the median value of alcohol intake of 12.5 kcal of ethanol/kg/day. We observed that below that value, which, for a mean body mass of 70 kg corresponds to 156 g of ethanol, ie ∼13 ethanol units of 13 g per day [38], alcohol intake was compensated for by a decrease in non-alcoholic intake and the total intake was close to normative values. Above that median value, the total calorie intake largely exceeded norms. Therefore, at relatively low doses, ethanol seems to have a negative effect on food energy intake, maintaining a balance in total energy supplies. By contrast, above ∼12.5 kcal of ethanol per kg per day, the balance is disrupted and the total intake is in excess, suggesting another mechanism modulating alcohol and food consumption.

### The Relation between Energy Intakes, Body Weight, Fat Mass and Metabolism

Previous research [38], [39] suggests that when the proportion of alcohol in the diet is high (∼50%), part of the energy contained in the diet is lost or wasted. Consistent with this, we found that AD subjects who drink large amounts of alcohol (>12.5 kcal/kg/day) had lower weight, BMI and FM than the subjects drinking lower amounts of alcohol (<12.5 kcal/kg/day). To avoid two potential biases – the first being the higher proportion of FM in women than in men, and the second being the higher proportion of women in the “low alcohol” group compared with “high alcohol group” – we calculated the differences in men and women and observed higher weight, BMI and FM in the “low alcohol” group than in the “high alcohol” group in men and a similar trend in women. Furthermore, we also observed a negative relation between alcohol supplies and BMI or FM in both genders. Addolorato and colleagues had previously observed that heavy drinking AD subjects evidenced a lower body weight and FM than social drinkers [40], [41]. Our results further support and extend this result by finding similar results within our AD subjects. It may also explain why alcohol-dependence has been linked to both increases and decreases in body weight.

We also observed that, in AD subjects, basal metabolism was larger than the theoretical values according to the Schofield equations. It decreased significantly after withdrawal. At T1, basal metabolism strongly correlated with the energy contained in alcoholic beverages. Our data are in keeping with those of Addolorato and colleagues that suggested that the high resting energy expenditure and the preferential utilization of lipids as energy substrates explained the low BMI and FM observed in heavy drinking AD subjects [42]. In support for a stimulation of lipid oxidation in heavily drinking AD subjects (over 100 g of alcohol/day), the group of Addolorato observed a recovery of FM during abstinence, that was complete after 3 months [42]. Consistent with these results, we observed a low RQ in drinking AD subjects, which suggests an increased lipid metabolism in drinking AD subjects, and this RQ increased significantly after withdrawal. Furthermore, we observed a significant increase in lipid intake after 1–2 weeks of abstinence, whereas carbohydrate and protein intakes remained unaffected. This suggests that the recovery of FM observed by Addolorato et al. after alcohol withdrawal is due both to a decrease in lipid oxydation and a selective increase in lipid intake during recovery.

The paradoxically negative relation between alcohol intake and BMI or FM may be due to ethanol-induced basal metabolism activation, which can be attributed to sympathic activation [43], microsomal MEOS induction [44], [45] and/or inducing mitochondrial metabolism [46], [47]. However, ethanol may also exert a negative effect on nutrient absorption by the gastrointestinal tract.

### The Relation between Peptides, Energy Intake and Metabolism

Finally, we examined the evolution of various orexigenic and anorexigenic peptides between the onset and the end of withdrawal. Leptin is an adipocyte-derived hormone that is essential for the regulation of food intake and body weight [48], [49]. As rising with the increase in adiposity, the physiological role of leptin was originally thought to signal the brain to inhibit food intake (anorexigenic effect) limiting therefore the body weight gain [48]. On the other hand, leptin levels fall rapidly in response to fasting [50] or caloric restriction [51]. These low leptin levels induce overfeeding, by increasing levels of orexigenic peptides and reducing levels of anorexigenic peptides, and suppressing energy expenditure [49]. The leptin level can therefore be considered as a sensor for energy balance [52]. Here, we showed that AD subjects exhibited higher leptin levels compared to the controls, which is consistent with data from previous researches [22], [53], [54]. Insulin, which is also a long-term moderator of energy metabolism, and which is considered as a satietogenic peptide, [28] was higher compared to controls, but only significantly higher at T2. Our results are the first to demonstrate that the anorexigenic peptides PYY and GLP-1 were significantly higher and lower, respectively, when compared to the controls. Conversely, the orexigenic peptide ghrelin was significantly lower, which is consistent with some [26], [55] but not all prior studies [56]. Altogether, except for the decrease in GLP-1 that should have a positive effect on appetite, the increases in leptin, insulin and PYY and the decrease in ghrelin might induce an anorexigenic state that contributes to the decrease in food intake observed in AD subjects. We observed a strong negative correlation between leptin and leptin/BMI and alcoholic, non-alcoholic and total energy intake. This observation suggests that leptin might exert a negative effect on energy intake, including alcohol. This is consistent with the classic role of leptin as a regulator of energy intake [28].

Notably, however, we observed a negative relation between serum leptin level and oxidative metabolism (Fig 4B) in AD subjects, which is inconsistent with the role of leptin as a stimulator of oxidative metabolism [57]. This observation may mean that ethanol is a stronger metabolic stimulator than leptin, and that this stimulation induces large decreases in fat mass. Hence, this may help explain why subjects drinking high ethanol quantities present high metabolism and low fat mass and leptin levels.

More studies are necessary to determine the level of alcohol intake where BMI and fat mass start to shift towards a decrease in alcoholic patients. Additionally, experimental examining the role of leptin in the context of alcohol dependence would be highly valuable to better ascertain the role of leptin in the management of alcohol consumption, i.e. in ob/ob or db/db mice subjected to high and low level alcohol.

### A Dual Model of Energy Intake Regulation in Alcohol-dependent Subjects

In line with our observations, we propose a model to explain the complex relation between alcohol intake and BMI or FM regulation. This model ascribes a central role to plasma leptin in the regulation of energy intake (Fig 5). In AD subjects drinking “low alcohol” quantities, overall, metabolism and lipid oxidation are not promoted. FM is increased or unaffected. An increase of most anorexigenic peptides including leptin, and a decrease in ghrelin are observed. The alcohol related energy intakes are compensated for by a decrease in food intake. In AD subjects drinking “high alcohol” quantities, overall and especially lipid metabolism is increased while FM is decreased. The leptin levels are decreased and, irrespective of changes in other appetite factors, food intake is not inhibited: the total energy intakes (alcohol + food) largely exceed norms.

This model provides an explanation to the dual relation that we observed between alcohol intakes and FM, depending on the quantities of alcohol intake. It also suggests that, in AD subjects, the loss of control of intake is not to be attributed solely to an effect of alcohol on the brain reward circuit [58]. It could also be due to a progressive metabolic adaptation, with a new allostatic balance, where the capacity of energy intake is greatly increased compared to norms. We suggest that this adaptation of energy balance contributes, aside from the effects of ethanol on the brain reward system, to the loss of control of alcohol intake classically observed in AD subjects [29].

## Supporting Information

Table S1 **Characteristics of AD subjects in study 1 according to alcohol type.**
(DOC)

Table S2 **Biological characteristics of AD subjects at the onset of withdrawal in study 2.**
(DOC)

Text S1 **Material and Methodology.**
(DOC)
