# Supplementary material for: The Loss of Metabolic Control on Alcohol Drinking in Heavy Drinking Alcohol-Dependent Subjects
Source: PLoS One. 2012 Jul 9;7(7):e38682. doi: 10.1371/journal.pone.0038682 (PMC3392266; doi:10.1371/journal.pone.0038682)
Supplement: Table S1 — Characteristics of AD subjects in study 1 according to alcohol type. (DOC) [file pone.0038682.s001.doc]

**Table S1: Characteristics of AD subjects in study 1 according to alcohol type**

|  | **Beer**  (n = 17) | **Wine**  (n = 20) | **Spirit**  (n = 16) | **Mix**  (n = 43) |
| --- | --- | --- | --- | --- |
|  |  |  |  |  |
| **Anthropomorphic parameters** |  |  |  |  |
| Gender (M/F) | 11/6 | 11/9 | 12/4 | 29/14 |
| Age (y) | 46 ± 11 | 54 ± 11 | 50 ± 11 | 47 ± 11 |
| Weight (kg) | 72.6 ± 16.9 | 72.1 ± 11.7 | 74.4 ± 14.4 | 74.7 ± 17.5 |
| BMI (kg/m^2^) | 24.4 ± 4.9 | 24.5 ± 4.2 | 24.6 ± 3.6 | 24.9 ± 4.9 |
| BM theoretical (kcal) | 1596 ± 254 | 1569 ± 203 | 1674 ± 242 | 1646 ± 264 |
|  |  |  |  |  |
| **Nutrient intakes (kcal/kg/day)** |  |  |  |  |
| Total | 51.0 ± 24.2 | 28.9 ± 13.8 ** | 34.9 ± 9.1 | 41.2 ± 19.3 |
| Proteins | 4.3 ±1.9 | 3.5 ± 1.5 | 3.0 ± 0.9 | 3.4 ± 1.6 |
| Lipids | 8.4 ± 6.3 | 7.5 ± 3.5 | 7.0 ± 2.8 | 6.8 ± 3.7 |
| Carbohydrates | 19.6 ± 11.2 | 9.0 ± 7.9 *** | 8.4 ±3.6 ** | 13.8 ± 7.0 * |
| Non-alcohol | 32.3 ± 18.3 | 19.9 ± 12.4 * | 18.4 ± 5.8 * | 24.1 ± 9.8 |
| Alcohol | 18.6 ±10.1 | 9.0 ± 3.6 *# | 16.9 ±6.4 | 17.2 ± 13.1 |

Values are means ± SD. * p < 0.05, ** p < 0.01, *** p < 0.001 (compared with “Beer”); # p < 0.05 (compared with “Mix”). All variables were compared using one-way ANOVA followed, if results were significant, by post-hoc Tukey’s tests.
