# Supplementary material for: The Loss of Metabolic Control on Alcohol Drinking in Heavy Drinking Alcohol-Dependent Subjects
Source: PLoS One. 2012 Jul 9;7(7):e38682. doi: 10.1371/journal.pone.0038682 (PMC3392266; doi:10.1371/journal.pone.0038682)
Supplement: Table S2 — Biological characteristics of AD subjects at the onset of withdrawal in study 2. (DOC) [file pone.0038682.s002.doc]

**Table S2**: Biological characteristics of AD subjects at the onset of withdrawal in study 2

|  | **NR** | **AD-T1**  (n = 24) |
| --- | --- | --- |
|  |  |  |
| AST (UI/l) | 6-33 | 76 ± 72 |
|  |  |  |
| ALT (UI/l) | 14-23 | 61 ± 44 |
|  |  |  |
| γ-GT (UI/l) | 7-50 | 183 ± 172 |
|  |  |  |
| Total bilirubin (mg/dl) | 0.3-1.2 | 0.9 ± 0.4 |
| TBP (g/dl) | 6.5-7.5 | 7.3 ± 0.7 |
|  |  |  |
| Albumin (g/dl) | 3.5-5.2 | 4.2 ± 0.5 |
|  |  |  |
| MCV (µm^3^) | 85-95 | 100 ± 6 |
|  |  |  |

Values are means ± SD. NR = Normal range; AST = aspartate aminotranferase, ALT = alanine aminotransferase, γ*-GT* = γ-glutamyl transpeptidase; TBP = Total blood protein; MCV = Mean corpuscular volume.
