# Supplementary material for: The Loss of Metabolic Control on Alcohol Drinking in Heavy Drinking Alcohol-Dependent Subjects
Source: PLoS One. 2012 Jul 9;7(7):e38682. doi: 10.1371/journal.pone.0038682 (PMC3392266; doi:10.1371/journal.pone.0038682)
Supplement: Text S1 — Material and Methodology. (DOC) [file pone.0038682.s003.doc]

# Text S1

1. ***Biochemical assessment in the serum***.

Fasting glucose and insulin assays were performed, in order to apply the Homeostatic Model Assesment (HOMA): three blood samples were taken at 5 min intervals [1,2]. The sampled arm was wrapped in an electric blanket to provide “arterialised” blood. Plasma glucose was measured by the glucose oxydase method (Beckman Glucose Analyser II*®*, Beckman Instruments, Fullerton, CA) and plasma insulin by chemiluminescence, using a double antibody immunoassay on the ADVIA *Centaur®* Immunoassay System (Siemens Healthcare Diagnostics, Eschborn, Germany). β-cell function (B) and insulin sensitivity (S) were calculated after averaging plasma insulin of the three samples to avoid errors due to peak insulin variations, and integrating glucose and insulin values in the HOMA model. Values are expressed as percentage of the values of a reference population (%B and %S respectively).

Total cortisol was measured using a competitive polyclonal immunoassay, comprising an electromagnetic separation step followed by electrochemiluminescence quantitation with the Elecsys 1010/2010 analyser*®* (Roche Diagnostics, Mannheim, Germany). Results are expressed in nanomoles per litre (nmol/l).

For leptin and GLP-1 measurements, plasma was obtained from samples collected on heparinized tubes containing dipeptidyl-peptidase IV (DPP-IV) inhibitor (Linco Research, MO, USA). Leptin was measured with an RIA kit for human leptin (Linco Research, MO, USA), as decribed by Balligand et al.[3]. Results are expressed as ng/ml. GLP-1 was measured by an enzyme linked immunosorbant assay (Linco Research, MO, USA)[4,5].

For ghrelin measurements, blood samples were taken in tubes containing EDTA. Plasmas were acidified by 5 % of a 1N HCL solution, and supplemented with 1% of a solution containing 1% of phenymethylsulfonyl fluoride in methanol. Ghrelin was assayed using an RIA kit (Linco Research, MO, USA) that measures the active form of ghrelin (with an octanoyl group on serine 3) [4]. Results are expressed in pg/ml.

For PYY measurements, blood samples were taken in dry tubes, after supplementation of 15 μl of DPP-IV inhibitor per ml (Linco Research, MO, USA). After centrifugation, plasma was supplemented with a solution of aprotinine to obtain a final concentration of 1μg/ml (Sigma Aldrich, Bornem, Belgium). PYY samples were assayed using an RIA kit that measures both 1-36 and 3-36 forms of the peptide (Linco Research, MO, USA) [6]. Results are expressed in pg/ml. All samples and standard curves were assayed as duplicates.

1. ***Body composition assessments.***

Body weight was measured by a beam scale with subjects wearing light clothes and no shoes. Height was measured using a wall mounted stadiometer. Body Mass Index (BMI) was computed as the ratio between body weight (kg) and the square of the subject’s height (m^2^). Fat Mass (FM) was calculated from bioelectric impedance measurement (BIA) with a specific device (BF306, Omron ®, Matsusaka, Japan) [7]. BIA that is proportional to total body water and fat-free mass (FFM) was calculated assuming that 73% of FFM is total body water. FM was computed as the difference between body weight and FFM.

1. ***Basal Metabolism***

Theoretical basal metabolism was calculated according to Schofield equations that take into account the gender, age and body weight of the subject [8]. The current basal metabolism was calculated from respiratory gas exchange, measured over a 30 min period at rest after an overnight fast, by continuous indirect calorimetry using an open-circuit ventilated hood system (Monitor MBM-100®, Deltatrac, Datex Instrumentarium, Helsinki, Finland). The respiratory quotient (RQ) was calculated by dividing the CO2 output by the O2 consumed.

1. ***Dietary recall interview.***

A 7-day dietary recall interview [9], was collected by two expert dieticians (DG and JD), using the hospital’s standardized semi-structured paper and pencil retrospective interview form, adapted to alcohol-dependent populations according to the principles of the time-line followback approach of Sobell and Sobell [10] . This method has been widely evaluated in various clinical and non-clinical populations concerning amount of alcohol drinks intakes [10–13]. This retrospective interview was preferred to diaries for several reasons : 1- heavy drinkers have a high nonparticipation rate in surveys; 2- forgetting to fill in diary increases with increasing consumption; 3- simple quantity-frequency questionnaires usually leads to great underestimation [14] in particular in AD subjects; 4-prospective methods as diet diaries often lead to underestimation of quantities, due either to under reporting or to diary induced under-eating [15].

Because questionnaire construction affects responses and questionnaires that present many probes about atypical drinking or eating result in reports of greater consumption by ̴~25% [16], we first had a three months pre-test period where the dieticians adapted the questionnaire to add new probes until no new questions arised.

In the approach of Sobell and Sobell the interviewer starts by asking what is drunk regularly in a normal week using a calendar. Then the interviewer asks whether there were any specific occasions where the subject has drunk more or less than usual. The exact quantities are evaluated using glasses to calculate the precise amount of each drink. Furthermore, the questions are posed in a redundant manner in order to double check the quality of the obtained data.

These principles were applied to our 7 days recall interviews where subjects were questioned meal by meal (e.g. “Do you usually take a breakfast, what do you usually eat for breakfast? what do you usually drink for breakfast?”), course by course, item by item (e.g. “Do you sometimes take a yogurt? you had'nt told me that you were taking a yogurt for breakfast 3 days a week?”). Probing allows recovery of many items not originally reported such as frequent addition to food (e.g. “How big was the piece of bread? How much butter did you add to your piece of bread?”). The accuracy of quantity estimation of each item for food, alcoholic and non-alcoholic beverages was improved by the use of photographs depicting dishes or glasses. Then the patients were asked whether there were any specific occasions during the week when they had any specific drink or meals. Then they were questionned on the habits concerning food and drink shopping (e.g. “Who usually buys food at home? How many yogurts do you buy each week? How many beers do you buy every week?) and then we checked whether the quantities matched the quantities that had been told during meals and drinking description.

The diet history interviews lasted for 1 to1.5 hours. The nutrient intake of all food items were then calculated using computerized tables (Diet Expert V2®, LogicAL systems, Liège, Belgium), adapted to the local food habits according to the food composition tables of Paul Lambin Institute, School of Nutrition, Brussels, Belgium [17]. The energy content of food was computed as follows: 4kcal/g of protein, 4 kcal/g of carbohydrate (or equivalent), 9 kcal/g of fat and 5.6 kcal/ml for ethanol (7 kcal/g for a density of 0.8). The two dieticians had first trained together both on interviewing of patients and coding of data, and in a second pre-test step compared the data they obtained in order to ensure good inter-rater reliability.

Reference List

1. Hermans MP, Levy JC, Morris RJ, Turner RC (1999) Comparison of insulin sensitivity tests across a range of glucose tolerance from normal to diabetes. Diabetologia 42: 678-687.

2. Hermans MP, Levy JC, Morris RJ, Turner RC (1999) Comparison of tests of beta-cell function across a range of glucose tolerance from normal to diabetes. Diabetes 48: 1779-1786.

3. Balligand JL, Brichard SM, Brichard V, Desager JP, Lambert M (1998) Hypoleptinemia in patients with anorexia nervosa: loss of circadian rhythm and unresponsiveness to short-term refeeding. Eur J Endocrinol 138: 415-420.

4. Cani PD, Montoya ML, Neyrinck AM, Delzenne NM, Lambert DM (2004) Potential modulation of plasma ghrelin and glucagon-like peptide-1 by anorexigenic cannabinoid compounds, SR141716A (rimonabant) and oleoylethanolamide. Br J Nutr 92: 757-761.

5. Knauf C, Cani PD, Perrin C, Iglesias MA, Maury JF et al (2005) Brain glucagon-like peptide-1 increases insulin secretion and muscle insulin resistance to favor hepatic glycogen storage. J Clin Invest 115: 3554-3563.

6. Delzenne NM, Cani PD, Daubioul C, Neyrinck AM (2005) Impact of inulin and oligofructose on gastrointestinal peptides. Br J Nutr 93 Suppl 1: S157-S161.

7. Deurenberg P, Andreoli A, Borg P, Kukkonen-Harjula K, de LA et al (2001) The validity of predicted body fat percentage from body mass index and from impedance in samples of five European populations. Eur J Clin Nutr 55: 973-979.

8. Schofield WN (1985) Predicting basal metabolic rate, new standards and review of previous work. Hum Nutr Clin Nutr 39 Suppl 1: 5-41.

9. Block G (1982) A review of validations of dietary assessment methods. Am J Epidemiol 115: 492-505.

10. Sobell LC, Sobell MB, Leo GI, Cancilla A (1988) Reliability of a timeline method: assessing normal drinkers' reports of recent drinking and a comparative evaluation across several populations. Br J Addict 83: 393-402.

11. Sobell LC, Toneatto T, Sobell MB, Schuller R, Maxwell M (1990) A procedure for reducing errors in reports of life events. J Psychosom Res 34: 163-170.

12. Sobell LC, Kwan E, Sobell MB (1995) Reliability of a drug history questionnaire (DHQ). Addict Behav 20: 233-241.

13. Maisto SA, Sobell MB, Sobell LC (1982) Reliability of self-reports of low ethanol consumption by problem drinkers over 18 months of follow-up. Drug Alcohol Depend 9: 273-278.

14. Day N, McKeown N, Wong M, Welch A, Bingham S (2001) Epidemiological assessment of diet: a comparison of a 7-day diary with a food frequency questionnaire using urinary markers of nitrogen, potassium and sodium. Int J Epidemiol 30: 309-317.

15. de Castro JM (2006) Varying levels of food energy self-reporting are associated with between-group, but not within-subject, differences in food intake. J Nutr 136: 1382-1388.

16. Campbell VA, Dodds ML (1967) Collecting dietary information from groups of older people. J Am Diet Assoc 51: 29-33.

17. Institut Paul Lambin. (2004) Table de composition des aliments 2004. Brussels, Belgium.
